# Impact of Autophagy in Oncolytic Adenoviral Therapy for Cancer

**DOI:** 10.3390/ijms18071479

**Published:** 2017-07-10

**Authors:** Hiroshi Tazawa, Shinji Kuroda, Joe Hasei, Shunsuke Kagawa, Toshiyoshi Fujiwara

**Affiliations:** 1 Center for Innovative Clinical Medicine, Okayama University Hospital, 2-5-1 Shikata-cho, Kita-ku, Okayama 700-8558, Japan; shinkuro@okayama-u.ac.jp; 2 Department of Gastroenterological Surgery, Okayama University Graduate School of Medicine, Dentistry and Pharmaceutical Sciences, Okayama 700-8558, Japan; skagawa@md.okayama-u.ac.jp (S.K.); toshi_f@md.okayama-u.ac.jp (T.F.); 3 Department of Orthopedic Surgery, Okayama University Graduate School of Medicine, Dentistry and Pharmaceutical Sciences, Okayama 700-8558, Japan; joe@md.okayama-u.ac.jp; 4 Minimally Invasive Therapy Center, Okayama University Hospital, Okayama 700-8558, Japan

**Keywords:** oncolytic adenovirus, autophagy, immunogenic cell death

## Abstract

Oncolytic virotherapy has recently emerged as a promising strategy for inducing tumor-specific cell death. Adenoviruses are widely and frequently used in oncolytic virotherapy. The mechanism of oncolytic adenovirus-mediated tumor suppression involves virus-induced activation of the autophagic machinery in tumor cells. Autophagy is a cytoprotective process that produces energy via lysosomal degradation of intracellular components as a physiologic response to various stresses, including hypoxia, nutrient deprivation, and disruption of growth signaling. However, infection with oncolytic adenoviruses induces autophagy and subsequent death of tumor cells rather than enhancing their survival. In this review, we summarize the beneficial role of autophagy in oncolytic adenoviral therapy, including the roles of infection, replication, and cell lysis. Numerous factors are involved in the promotion and inhibition of oncolytic adenovirus-mediated autophagy. Furthermore, recent evidence has shown that oncolytic adenoviruses induce autophagy-related immunogenic cell death (ICD), which enhances the antitumor immune response by inducing the activation of danger signal molecules and thus represents a novel cancer immunotherapy. Understanding the precise role of oncolytic adenovirus-induced autophagy and ICD could enhance the therapeutic potential of oncolytic adenoviral therapy for treating various cancers.

## 1. Introduction

Oncolytic virotherapy has emerged as a novel antitumor strategy for inducing the lytic death of tumor cells as a result of rapid viral replication [1]. Although a number of different viruses are used in virotherapy, including adenoviruses, herpes simplex virus, measles virus, reovirus, and Newcastle disease virus, adenovirus serotype 5 (Ad5) is one of the most commonly used in oncolytic virotherapy [1]. Oncolytic adenoviruses are internalized by target cells via binding to the Coxsackie and adenovirus receptor (CAR) on the cell surface. As both normal and tumor cells express CAR within tumor-affected tissues, oncolytic adenoviruses are biogenetically modified to exhibit tumor-specific replication capability. Virus replication is regulated primarily by the induction of viral gene expression within the target cells. In adenovirus replication, induction of the early *E1* gene is the most critical factor, as *E1*-deletion adenoviruses cannot replicate. Therefore, the wild-type *E1* promoter has been biogenetically modified using several different tumor-specific promoters, including the human telomerase reverse transcriptase (hTERT) [2,3,4,5,6], midkine [7,8], cyclooxygenase-2 [9], and survivin [10] promoters. As hTERT is reportedly overexpressed in >80% of malignant tumor tissues [11], the *hTERT* promoter is a useful tool for tumor-specific regulation of viral gene expression [12]. Tumor-specific, promoter-regulated oncolytic adenoviruses are therefore ideal vectors for inducing tumor cell death through viral replication.

The mechanism of oncolytic adenovirus-mediated tumor suppression involves virus-induced activation of the autophagic machinery in tumor cells [13]. Autophagy produces energy through lysosomal degradation of cytoplasmic cellular components in autophagosomes [14]. Several physiologic conditions, including nutrient deprivation [15], hypoxia [16], and disruption of growth signaling [17], activate the autophagic machinery to enhance cell survival. However, some pathogenic viruses and bacteria also induce autophagy in infected cells [18,19]. Virus-induced autophagy plays both anti-viral defense and pro-viral replication roles in infected cells [18]. Several oncolytic adenoviruses are known to induce autophagy; in tumor cells, this process leads to cell death rather than survival [10,20,21]. For example, we generated a telomerase-specific, replication-competent oncolytic adenovirus, OBP-301, which drives the adenoviral *E1A* and *E1B* genes under control of the *hTERT* promoter for tumor-specific viral replication, and we found that OBP-301 induces lytic death of tumor cells with telomerase activity [6]. OBP-301-mediated autophagy induction is strongly associated with decreased viability of tumor cells [22,23]. Thus, induction of autophagy plays a crucial role in oncolytic adenovirus-mediated tumor suppression.

Recent evidence suggests that antitumor therapy-induced autophagy is associated with immunogenic cell death (ICD), which involves the induction of antitumor immune responses via the release of damage-associated molecular pattern (DAMP) molecules and tumor-associated antigens (TAAs) [24]. DAMP molecules include adenosine triphosphate (ATP), high-mobility group B1 (HMGB1), calreticulin (CRT), and uric acid [25]. A variety of ICD inducers are used in antitumor therapy, including chemotherapeutic agents, irradiation, and photodynamic treatment [26]. Oncolytic adenoviral therapy is also hypothesized to induce ICD via the induction of autophagy and the activation of DAMP molecules and TAAs [26,27]. Moreover, oncolytic virotherapy induces the release of pathogen-associated molecular pattern molecules, which function as another type of danger signal [27,28,29]. Therefore, oncolytic adenovirus-induced autophagy could function as both an antitumor effector and antitumor immune stimulator in oncolytic virotherapy.

In this review, we summarize the beneficial role of autophagy in oncolytic adenovirus-mediated tumor suppression processes, including virus infection, replication, and cell lysis. Factors that modulate autophagy are also described in the context of oncolytic adenoviral therapy. Moreover, the preclinical and clinical relevance of autophagy-mediated ICD in oncolytic adenoviral therapy are discussed in terms of antitumor immunotherapy.

## 2. Role of Autophagy in Oncolytic Adenoviral Therapy

A number of different oncolytic adenoviruses have been shown to induce autophagy in tumor cells. The relationship between the type of oncolytic adenovirus and role of autophagy in the cell is summarized in our previous review [13]. A variety of oncolytic biogenetically modified adenoviruses encoding tumor-specific promoters and/or modified fiber knobs have been developed to induce autophagy and subsequent tumor cell death through enhanced viral replication and infectivity. By contrast, oncolytic adenoviruses encoding the wild-type *E1* promoter and intact fiber knobs induce only a mild form of autophagy that promotes the survival of tumor cells. Thus, the intracellular level of autophagy is a crucial factor in determining whether oncolytic adenoviral therapy leads to tumor cell death or survival.

**Figure 1:**
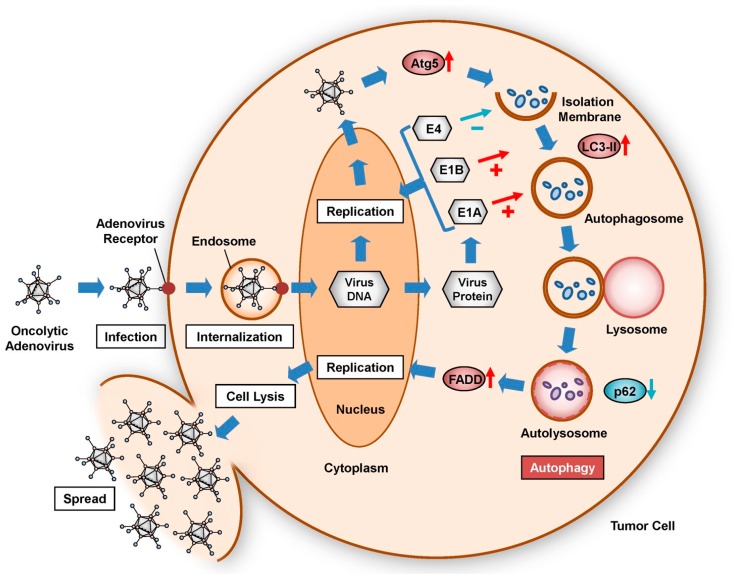
Life cycle of oncolytic adenoviruses and induction of autophagy in infected tumor cells**.** Oncolytic adenoviruses are internalized into the cytoplasm through binding to adenovirus receptors and subsequent endosomal encapsulation. Virus DNA is transferred into the nucleus, resulting in the replication of oncolytic adenoviruses. During viral replication, adenoviral DNA-derived E1A, E1B, and E4 accumulate in the cytoplasm. Expression of Atg5 is upregulated in response to viral replication. After the Atg5–Atg12 complex binds to the isolation membrane, LC3-II, p62, and intracellular components cooperatively accumulate at the isolation membrane, resulting in formation of an autophagosome. The autophagosome fuses with the lysosome to become an autolysosome, in which p62 and cytoplasmic components are degraded under acidic conditions. FADD-induced enhancement of autophagy contributes to viral replication, cell lysis, and virus spread.

The intracellular level of autophagy increases in tumor cells during the life cycle of oncolytic adenoviruses (Figure 1). To infect tumor cells, oncolytic adenoviruses bind to primarily to CAR on the surface of target cells. Modification of the adenovirus fiber knob improves infectivity. Following attachment to target cells, oncolytic adenovirus particles are internalized by encapsulation in endosomes. After endosomal lysis and release into the cytosol, viral genomic DNA is delivered to the nucleus via intracellular trafficking. The oncolytic adenovirus replicates in the nucleus through the synthesis of virus DNA using the cell’s machinery. Modification of the wild-type *E1* promoter to a tumor-specific promoter enhances the replication rate of oncolytic adenoviruses in target tumor cells. Replication of the oncolytic adenovirus within the tumor cell in turn contributes to the induction of autophagy.

Several adenovirus proteins, including E1A, E1B, and E4, are expressed and accumulate in the cytoplasm of target cells during viral replication (Figure 1). Oncolytic adenovirus-induced autophagy is promoted by E1A and E1B but suppressed by E4 (Figure 1). The differential induction of autophagy in tumor cells can be illustrated by three types of adenoviruses exhibiting different E1A and E1B status (wild-type Ad5, E1B-deleted Adhz60, and E1A- and E1B-deleted AdlacZ). Of these viruses, wild-type Ad5 induces the strongest autophagy, whereas E1A- and E1B-deletion AdlacZ induces only minimal autophagy [30]. By comparison, E4 suppresses autophagy through activation of the anti-autophagic mammalian target of rapamycin (mTOR) signaling pathway [31] and inhibition of pro-autophagic unc-51-like autophagy-activating kinase 1 (ULK1) activity [32]. E4 inhibits the antitumor potential of the E1B-55K-deletion oncolytic adenovirus ONYX-015 [33]. Thus, oncolytic adenovirus-associated proteins can modulate the level of autophagy.

The expression of various autophagy-related biomarker proteins is associated with induction of the autophagic machinery by oncolytic adenoviruses, such as autophagy related 5 (Atg5) [34], microtubule-associated protein 1, light chain 3 (LC3) [35], and p62 [36] (Figure 1). After replication of oncolytic adenoviruses, Atg5 is upregulated in infected tumor cells [21]. Atg5 and Atg12 form a complex that accumulates at the isolation membrane. After conversion of the long form of LC3-I to the short form, LC3-II, LC3-II and p62 cooperatively bind to the isolation membrane, which results in the formation of an autophagosome containing intracellular organelles. The autophagosome then fuses with a lysosome to become an autolysosome, or acidic vesicular organelle (AVO), which degrades p62 and the intracellular organelles under acidic conditions. The intracellular level of autophagy in oncolytic adenovirus-infected tumor cells can be evaluated by analyzing changes in autophagy-related biomarkers, such as upregulation of Atg5 and LC3-II, p62 downregulation, and the formation of cytoplasmic AVOs. In addition, activation of the Fas-associated via death domain (FADD)/caspase-8 signaling pathway following autophagy induction has been shown to enhance oncolytic adenovirus-mediated autophagy [37], resulting in autophagic cell death via interaction with Atg5 and FADD [38]. These autophagy-related biomarkers are useful for assessing the intracellular level of autophagy in oncolytic adenoviral therapy.

When extensive autophagy is induced following rapid viral replication, oncolytic adenoviruses primarily induce the lysis of tumor cells, resulting in further virus spread (Figure 1). As extensive autophagy induces cell lysis, autophagy-inducing and -inhibiting strategies can be employed to direct the life and death of target tumor cells in oncolytic adenoviral therapy. Next, we will focus on the factors that promote or inhibit the induction of oncolytic adenovirus-mediated autophagy.

## 3. Factors that Promote or Inhibit Oncolytic Adenovirus-Mediated Autophagy

**Figure 2:**
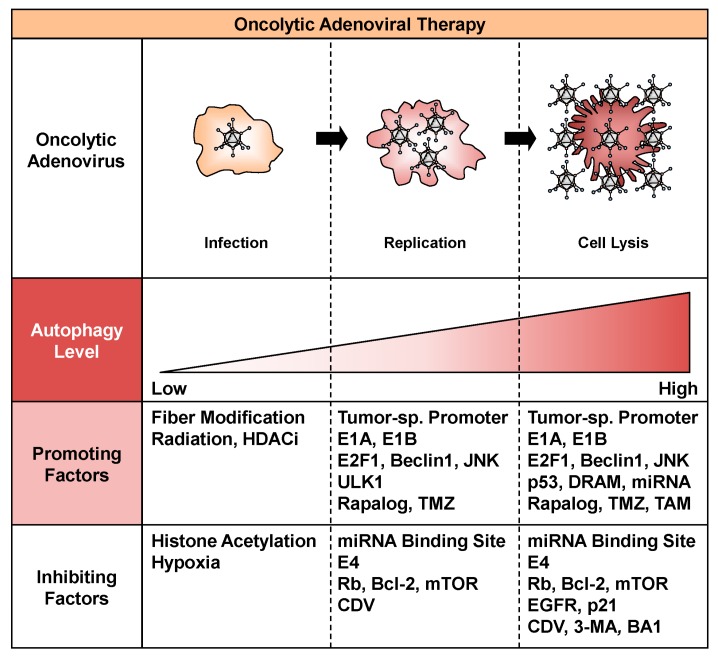
Factors that promote or inhibit oncolytic adenovirus-mediated autophagy. Factors that promote or inhibit oncolytic adenovirus-mediated autophagy are shown. HDACi, histone deacetylase inhibitor; JNK, c-Jun N-terminal kinase; ULK1, unc-51-like autophagy-activating kinase 1; TMZ, temzolomide; miRNA, microRNA; mTOR, mammalian target of rapamycin; CDV, cidofovir; DRAM, damage-regulated autophagy modulator; TAM, tamoxifen; EGFR, epidermal growth factor receptor; 3-MA, 3-methyladenine; BA1, bafilomycin A1.

A variety of factors can promote autophagy during the process of oncolytic adenoviral therapy, including infection, replication, and cell lysis (Figure 2). The efficacy of oncolytic adenoviruses in infecting target cells is one of the most important issues to consider for increasing the intracellular level of autophagy. Infection with adenoviruses depends primarily on the level of CAR expression on the surface of target cells. Upregulation of CAR expression enhances the infectivity of oncolytic adenoviruses, leading to extensive autophagy. For example, ionizing radiation increases the expression of CAR on the surface of tumor cells [39]. In addition, several histone deacetylase inhibitors, such as trichostatin A, sodium phenylbutyrate, FK228, and FR901228, increase the expression of CAR [40,41,42]. By contrast, modification of the fiber knob improves the infectivity of oncolytic adenoviruses independent of CAR expression. For example, incorporation of RGD peptide enhances binding to integrins αVβ3 and αVβ5. The RGD fiber-modified hTERT-driven OBP-301 variant (OBP-405) induces more profound autophagic death of malignant brain tumor cells than OBP-301, which encodes the wild-type fiber [43]. Fiber modification with the polylysine PK7 motif increases the affinity for heparin sulfate proteoglycans. PK7 fiber-modified survivin promoter-driven CRAd-S-pk7 induces autophagic death of malignant brain tumor cells in combination with temzolomide (TMZ) [44]. Moreover, a chimeric fiber knob composed of adenovirus serotype 3 (Ad3) and Ad5 fibers binds to CD46 on the cell surface. Ad5/3 fiber-modified human chorionic gonadotropin (hCG)-expressing Ad5/3∆24hCG, which lacks a 24-bp segment (919-943) in the *E1A* region, induces autophagic death of human cancer cells [45]. Enhanced infectivity contributes to the high uptake of oncolytic adenoviruses and induction of virus-mediated autophagy in tumor cells.

The replication of oncolytic adenoviruses also affects the intracellular level of autophagy. Modification of the wild-type *E1* promoter is a promising strategy for improving the level of viral replication because the expression level of E1 is crucial for the replication of oncolytic adenoviruses. The tumor-specific promoter enhances the viral replication level only in tumor cells, without affecting normal cells. The *hTERT* promoter is one of the most useful tools for inducing tumor-specific cell death. For example, a conditionally replicating oncolytic adenovirus, hTERT-Ad, in which a 255-bp *hTERT* promoter fragment is inserted into the wild-type *E1A* promoter region, induces the autophagic death of malignant brain tumor cells [20]. OBP-301, which contains a 455-bp *hTERT* promoter, induces the autophagic death of a variety of tumor cells with telomerase activities [22,23]. Moreover, the *hTERT* promoter-driven oncolytic adenovirus OBP-301 can replicate more efficiently than wild-type Ad5, even in hypoxic tumor microenvironments [46].

The adenoviral proteins E1A and E1B play roles in the induction of pro-autophagic signaling pathways. Adenoviral E1A binds to the tumor suppressor Rb, resulting in release of the transcription factor E2F1 from the Rb-E2F1 complex [47]. E2F1 activation induces autophagy via the upregulation of autophagy-related proteins, including Atg5 and LC3, in transactivation-dependent and -independent manners [48,49]. By contrast, adenoviral E1B interacts with pro-autophagic Beclin1, resulting in dissociation of the Beclin1-Bcl-2 complex and the induction of Beclin1-dependent autophagy [50]. During replication of the oncolytic adenovirus Delta-24-RGD, activation of the c-Jun N-terminal kinase pathway is also involved in suppression of Beclin1-Bcl-2 complex formation via the phosphorylation of Bcl-2 [51]. Moreover, it has been shown that suppression of the mTOR signaling pathway by rapalogs such as rapamycin and everolimus enhances autophagic cell death in oncolytic adenoviral therapy [43,52]. TMZ has been shown to enhance oncolytic adenovirus-induced autophagic death of malignant tumor cells [44,53,54]. Tamoxifen enhances the antitumor effect of survivin-driven CRAd-S-5/3 in primary malignant brain tumor cells [55]. In addition, adenovirus infection has been shown to induce accumulation of the sphingolipid metabolite ceramide, which is associated with autophagy-related cell death [56,57]. As sphingolipid metabolism is proposed to be a key regulator in autophagy induction [58], ceramide-inducing reagents, such as anti-folate pemetrexed and the sphingosine-1-phosphate receptor modulator FTY720, may enhance oncolytic adenovirus-mediated autophagy [57]. Adenoviral E1 and autophagy-inducing reagents efficiently activate the autophagic machinery in oncolytic virotherapy.

The activation of therapeutic transgenes is another useful strategy for enhancing the antitumor effect of oncolytic adenoviruses through the induction of autophagy. Although there are many types of therapeutic transgenes, those that specifically induce autophagy are the best candidates for enhancing antitumor effects in oncolytic adenoviral therapy. For example, activation of pro-autophagic Beclin-1 expression by Beclin-1-armed oncolytic adenoviruses enhances the autophagic death of malignant tumor cells [59]. In addition, the tumor suppressor gene *p53* is a multifunctional transcription factor that regulates diverse cellular processes, including autophagy, for tumor suppression [60]. We generated a *hTERT* promoter-driven OBP-301 variant (OBP-702) that expresses *p53* [61,62]. OBP-702 exhibited a more profound autophagy-associated antitumor effect than OBP-301 [63]. OBP-702 induced significant autophagy by inducing expression of the pro-autophagic protein damage-regulated autophagy modulator and suppressing expression of the anti-autophagic factor p21 [63]. We demonstrated the involvement of E2F1-regulated microRNAs (miRNAs) in oncolytic adenovirus-induced autophagic cell death [23,63]. E2F1-mediated activation of *miR-7* is involved in OBP-301-mediated autophagic death of human lung cancer cells through suppression of the anti-autophagic factor, epidermal growth factor receptor [23]. E2F1-mediated activation of *miR-93* and *miR-106* suppresses the expression of anti-autophagic p21 in OBP-702-mediated autophagy in human osteosarcoma cells [61,63]. These findings suggest that p53 and E2F1-regulated miRNAs are crucial factors that must be considered for fine-tuning oncolytic adenovirus-induced autophagy.

A number of factors can also inhibit autophagy by suppressing the infection, replication, or autophagy-inducing activity of oncolytic adenoviruses (Figure 2). Low infectivity of oncolytic adenoviruses results in poor induction of autophagy. CAR-negative tumor cells are highly resistant to oncolytic adenovirus-mediated antitumor effects. Downregulation of CAR expression by histone acetylation in the *CAR* gene promoter attenuates the infectivity of oncolytic adenoviruses for tumor cells [64]. The presence of a hypoxic tumor microenvironment also suppresses the expression of CAR, resulting in low infectivity [65]. In addition, a low rate of oncolytic adenovirus replication can suppress the induction of autophagy. The insertion of a miRNA binding site was recently reported as a means of suppressing the replication and cytotoxic effects of oncolytic adenoviruses in normal tissue. For example, insertion of the miR-122 binding site into the 3’-untranslated region of the gene encoding E1 effectively attenuates the replication of oncolytic adenoviruses in liver tissue, in which miR-122 is highly expressed [66,67,68]. This strategy is very useful for circumventing the hepatotoxicity of oncolytic adenoviruses. By contrast, we previously reported that cidofovir, an antiviral compound approved for the clinical treatment of adenovirus infection [69], inhibits the antitumor effect of hTERT-driven OBP-301 by suppressing viral replication [70]. Adenoviral E4 suppresses autophagy through activation of the mTOR signaling pathway [31] and subsequent inhibition of ULK1 activity [32] during viral replication. Oncolytic adenovirus-mediated autophagy can also be inhibited by directly suppressing the autophagic machinery. For example, 3-methyladenine, an inhibitor of pro-autophagic phosphatidylinositol 3-kinase class III, inhibits the induction of autophagy associated with oncolytic adenoviruses through suppression of both the autophagy signaling pathway and viral replication [30]. Bafilomycin A1, an inhibitor of lysosomal function, inhibits the induction of autophagy and antitumor effects of oncolytic adenoviruses by preventing fusion of the autophagosome and lysosome [37]. Thus, the life cycle of oncolytic adenoviruses is a key target that can be exploited to suppress oncolytic adenovirus-mediated autophagy.

## 4. Autophagy-Mediated Immunogenic Cell Death in Oncolytic Adenoviral Therapy

**Figure 3:**
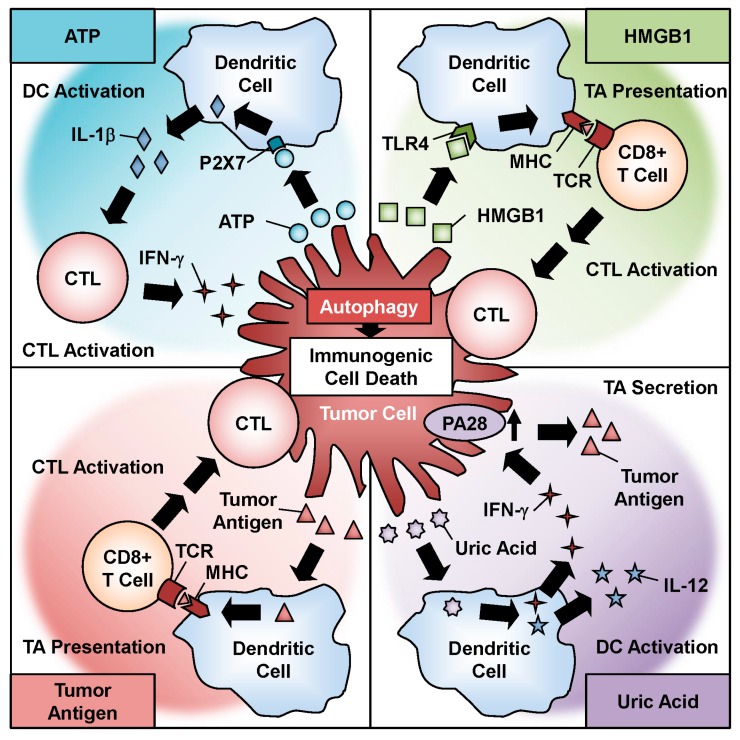
Role of oncolytic adenovirus-induced immunogenic cell death in antitumor immunity. Oncolytic adenovirus-induced autophagy contributes to the induction of immunogenic cell death, which causes the release of danger signal molecules, such as ATP, HMGB1, uric acid, and tumor antigens.

Recent evidence has shown that chemotherapeutic agents induce ICD in association with programmed cell death pathways such as apoptosis, necrosis, and autophagy [24]. ICD is a cellular phenomenon in which an antitumor immune response is induced through the activation of intracellular factors such as DAMP molecules and TAAs. Oncolytic adenoviruses have been shown to induce ICD with autophagy and the activation of a number of DAMP molecules and TAAs [27,28,29] (Figure 3). Recent studies have also suggested a functional role for the autophagic machinery in the immune system [71]. The first DAMP molecule to consider is ATP, which is the primary energy currency of cellular metabolism. ATP is actively secreted by tumor cells after infection. Ad5/3-D24-GM-CSF, an Ad5/3 fiber-modified oncolytic adenovirus armed with granulocyte macrophage colony-stimulating factor (GM-CSF), induces ATP secretion in prostate cancer cells after infection [53]. Moreover, treatment of tumor cells with the combination of TMZ and Ad5/3-D24-GM-CSF enhances ATP secretion by tumor cells by increasing the intracellular level of autophagy [53]. The active secretion of ATP is thought to be associated with autophagy and lysosomal exocytosis in dying cells [72,73]. Extracellular ATP serves as a danger signal that activates dendritic cells (DCs) by binding to the P2X7 receptor. ATP-stimulated DCs secrete IL-1β, which polarizes IFN-γ-producing cytotoxic T lymphocytes (CTLs) [24]. In the presence of IFN-γ, interaction of FADD with Atg5 induces autophagic cell death by activating caspase-8 [38]. In addition, excessive autophagy enhances apoptosis induction in response to Fas ligand [74], which is an important pathway for killing by CTLs.

The non-histone nuclear factor HMGB1, the most abundant non-histone protein in the nucleus, is another DAMP molecule. HMGB1 is passively released from tumor cells after infection. Ad5/3-D24-GM-CSF induces the release of HMGB1 as well as ATP in virus-infected tumor cells [53]. The release of HMGB1 is reportedly correlated with autophagy induction in antitumor therapy [75]. Extracellular HMGB1 activates DCs by binding to toll-like receptor 4, resulting in the activation of CTLs. A recent clinical study of oncolytic adenoviral therapy in 202 cancer patients showed that a low serum HMGB1 level at baseline is a useful predictive biomarker [76]. These data suggest that oncolytic adenovirus-induced ICD with HMGB1 release activates the antitumor immune response more effectively in patients with low HMGB1 levels compared with patients with high HMGB1 levels. 

CRT is a DAMP molecule that functions as a chaperone in the endoplasmic reticulum (ER). During apoptotic ICD induction, ecto-CRT is translocated from the cytoplasmic ER to the cell surface [26]. Although oncolytic adenoviruses also induce tumor cells to become ecto-CRT positive [53], whether autophagy is associated with ecto-CRT induction remains to be elucidated. 

Uric acid, which is the primary end metabolite of purine catabolism, is another DAMP molecule. Uric acid is passively released from tumor cells after infection. We demonstrated that OBP-301 causes lytic cell death with the release of uric acid, which results in the activation of DCs to produce high amounts of IFN-γ and IL-12 [22]. Moreover, IFN-γ-mediated upregulation of the proteasome activator PA28 in tumor cells contributes to the release of TAAs. TAAs function as immunogenic stimulators in cooperation with DAMP molecules. A recent report suggested that oncolytic adenovirus-induced autophagy is responsible for the presentation of TAAs incorporated into adenoviral capsids in a major histocompatibility complex (MHC) class II-dependent manner [77]. Moreover, it has been shown that autophagy facilitates the generation of TAAs in a MHC class I-dependent manner in the presence of IFN-γ, resulting in cytolysis of CTLs [78]. Thus, oncolytic adenovirus-induced autophagy and ICD could enhance the antitumor immune response by priming immune cells via DAMPs, TAAs, and cytokines.

## 5. Conclusions

Oncolytic adenoviral therapy is a promising strategy for inducing tumor-specific cell death via the activation of autophagy. Recent reports have demonstrated the beneficial role of autophagy in oncolytic adenoviral therapy. Persistent and extensive autophagy induced by oncolytic adenoviruses plays a crucial role in the death of tumor cells. Stimulation of CAR expression, fiber modification, insertion of tumor-specific promoters, induction of therapeutic transgenes, and the use of autophagy-inducing reagents are useful strategies for inducing extensive autophagy through enhancement of viral infection, replication, and cell lysis. However, the precise roles of oncolytic adenovirus-induced autophagy and ICD in antitumor immunity remain to be elucidated. Therefore, exploring the functional role of oncolytic adenovirus-induced autophagy and ICD could improve the therapeutic potential of oncolytic adenoviral anticancer therapy.

## Abbreviations

Ad5	Adenovirus serotype 5
CAR	Coxsackie and adenovirus receptor
hTERT	Human telomerase reverse transcriptase
ICD	Immunogenic cell death
DAMP	Damage-associated molecular pattern
TAA	Tumor-associated antigen
ATP	Adenosine triphosphate
HMGB1	High-mobility group protein B1
CRT	Calreticulin
mTOR	Mammalian target of rapamycin
ULK1	unc-51-like kinase 1
Atg5	Autophagy-related 5
LC3	Microtubule-associated protein 1 light chain 3
AVO	Acidic vesicular organelle
FADD	Fas-associated via death domain
TMZ	Temzolomide
Ad3	Adenovirus serotype 3
hCG	Human chorionic gonadotropin
miRNA	microRNA
GM-CSF	Granulocyte macrophage colony-stimulating factor
DC	Dendritic cell
CTL	Cytotoxic T lymphocyte
ER	Endoplasmic reticulum
